# The photodynamic inactivation in the control of the seafood zoonotic parasite, *Anisakis* sp.

**DOI:** 10.1007/s00436-026-08635-z

**Published:** 2026-03-06

**Authors:** P. Ramos, A. S. Joaquinito, M. M. Oliveira, M. G. P. M. S. Neves, A. Almeida, M. A. F. Faustino

**Affiliations:** 1 IPMA, I.P., Portuguese Institute for the Sea and Atmosphere, Av. Doutor Alfredo Magalhães Ramalho, nº6, 1495-165, Algés, Portugal; 2 CIIMAR/CIMAR LA, University of Porto. Interdisciplinary Centre of Marine and Environmental Research., University of Porto - Terminal de Cruzeiros do Porto de Leixões, Av. General Norton de Matos S/N, 4450-208 Matosinhos, Portugal; 3 LAQV–REQUIMTE and Department of Chemistry, University of Aveiro, Campus Universitário de Santiago, 3810–193 Aveiro, Portugal; 4 Lusófona University, ECEO, Campo Grande 376, 1749-024 Lisboa, Portugal; 5 Department of Biology and CESAM, University of Aveiro, Campus Universitário de Santiago, 3810-193 Aveiro, Portugal

**Keywords:** L3 *Anisakis* larvae, Anisakiasis, Photodynamic inactivation, Porphyrin, Gut destruction, Zoonotic parasite control

## Abstract

*Anisakis* spp. L3 larvae commonly found in marine fish species are an important biological hazard when raw or inadequately thermally processed fishery products are consumed with live larvae, highlighting the need for effective control strategies. This study evaluated the efficacy and mechanism of photodynamic inactivation (PDI) of *Anisakis* L3 larvae, using the cationic photosensitizer (PS) 5,10,15,20-tetrakis(1-methylpyridinium-4-yl)porphyrin tetraiodide (TMPyP). Larvae were collected from Atlantic horse mackerel and grouped by size: batch A (larvae ≈ 1.5 cm) and batch B (larvae ≈ 2.0 cm). Inactivation assays were performed in microcosms at 50 µM of TMPyP under different conditions: incubation times (15 min to 21 h); incubation temperatures (0.5 and 22 °C); irradiation period (2–8 h) and light irradiance (4, 14 and 67 mW/cm^2^). Viability and tissue damage of the larvae were assessed at post-treatment. In both batches, 100% of larvae mortality was achieved after 21 h of dark incubation at 22 °C with TMPyP, followed by 6–8 h of white light irradiation at 67 mW/cm^2^. Similar results were observed in batch B, when the dark incubation was performed at 0.5 °C. Notably, batch B, larvae showed more exuberant cytotoxic effect, including severe damage of the intestinal epithelium, leading to loss of the characteristic triradiate appearance. These findings indicate that PDI using TMPyP is effective in inactivating *Anisakis* larvae and could be a potential alternative to freezing for ensuring the safety of fishery products in the food industry.

## Introduction

The third-stage (L3) larvae of *Anisakis* spp. (Nematoda: Anisakidae) are commonly found in a high number of commercial valuable fish species due to their low host specificity. Their presence is often underestimated in a great variety of processed fishery products (e.g. stacks, fillets and canned fish) (Ramos 2020, 2022a). Nowadays, the presence of L3 *Anisakis* larvae in fishery products is a significant concern for all stakeholders in value chain, including food business operators, competent authorities and consumers. Besides causing economic losses they also pose a serious threat to the consumer’s health. Viable L3 larvae are an important biological hazard associated with the consumption of wild marine fish or squids, particularly when they are consumed raw or are inadequately thermally processed, such as in marinated or salted fishery products (Commission Regulation (EU) N.º 1276/2011). L3 larvae of *A. simplex* s.s., *A. pegreffii* and *A. typica* can lead to the human zoonotic disease, anisakiasis (EFSA BIOHAZ Panel, 2024a). This foodborne disease is most frequently reported in Asian countries where raw fish consumption is common. However, the disease is becoming emergent in European countries due to the tendency of consuming raw fish dishes, such as sushi, sashimi and brined fish (Ramos et al. 2011; Robertson 2018). The invasive form of the anisakiasis can have four clinical types: gastric, intestinal, ectopic and IgE-mediated allergic reactions with or without digestive symptoms (Audicana et al. 2008). The allergic reactions range from urticarial and angioedema to life-threatening anaphylactic shock (Nieuwenhuizen 2016). Furthermore, it is believed that reported cases are underestimated, with only those requiring hospital support being reported (Mladineo 2019). Although anisakiasis usually resolves spontaneously with the detachment and expulsion of the larvae, since the larvae cannot reach the adult stage in the human intestinal tract and dies in a few days, the endoscopic extraction of the larvae is the used treatment for gastric and intestinal anisakiases. Despite antibiotics, anticholinergics, corticosteroids, anti-helminthic albendazole (Arias-Diaz et al. 2006) and ivermectin (Polak et al. 2020) have been proposed to treat anisakiasis, there are not effective pharmacological treatments against *Anisakis* larvae (Anza et al. 2021). The standard method for preventing anisakiasis involves freezing fish prior to its consumption in raw or lightly preserved forms. This is in accordance with European legislation, transposed into Portuguese law, which aims to prevent viable *Anisakis* larvae (L3) from reaching the consumer. Furthermore, as a preventive measure, educational strategies have been implemented targeting stakeholders in the food chain (Ramos 2020). At EU level, Regulation (EC) No 853/2004 stated that food business operators must carry out a visual examination of fishery products to detect the presence of visible parasites and prevent clearly contaminated fishery products, such as fillets, are not placed on the market for human consumption. However, the effectiveness of the visual inspection methods, particularly candling, depends on factors such as the colour, texture and thickness of the fillets, as well as of the operator’s experience (Levsen and Lunestad 2005). These limitations increase the risk of viable *Anisakis* larvae reaching consumers. The Commission Regulation (EU) No 1276/2011 stated that fishery products intended to be consumed raw, marinated, salted or otherwise treated, if the processing is insufficient to kill nematode larvae, must be frozen at least − 20 ˚C for not less than 24 h or − 35 ˚C for not less than 15 h in all parts of the product in order to inactivate *Anisakis* larvae or alternatively, heat treatment at 60 ˚C or higher for at least one minute. Despite freezing process can devitalize larvae, it may under certain conditions affects the sensorial characteristics of marinated or slightly salted fishery products (Giarratana et al. 2014). This fact can lead to the deliberate consumption of raw fish without prior freezing, thereby increasing the risk of disease. Another physical treatment, high-pressure processing (HPP) used for raw treating fish, causes changes in muscle colour and appearance which limits its application (Brutti et al. 2010). However, it is suitable for certain fish species or specific processed fish products (EFSA BIOHAZ Panel 2024b). In this context, there is an urgent need to develop new methods or drugs that can effectively make *Anisakis* L3 larvae non-viable.

Natural products, such as plant extracts, essential oils and compounds isolated from them have been studied as alternatives to freezing/heating for *Anisakis* inactivation, when used as additives in raw food preparation. Their nematocidal activity has been attributed to the individual or synergistic actions of their active components. Saline solutions of plant extracts of *Perilla frutescens* also known as Korean perilla (Kasuya et al. 1988, 1990); turmeric, *Curcuma longa* (Suzuki et al. 1994); ginger, *Zingiber officinale* (Goto et al. 1990; Lin et al. 2010) and Ethiopian flora (Anza et al. 2021) have been shown to exert activity against *Anisakis* larvae. Essential oils of chamomilla, *Matricaria chamomilla* (Romero et al. 2012); sharp-leaf galangal, *Alpinia oxyphylla* (Lin et al. 2013); thyme, *Thymus vulgaris* (Giarratana et al. 2014); tea tree, *Melaleuca alternifolia* (Gómez-Rincón et al. 2014); peppermint, *Mentha piperita* (Romero et al. 2014); perillaldehyde, the main component of *Perilla frutescens* (Valero et al. 2015); nutmeg, *Myristica fragans* (López et al. 2015); catmint, *Nepeta cataria* (Giarratana et al. 2017b); ar-turmerone isolated from *Curcuma longa* (Valero et al. 2015); wild marigold, *Tagetes minuta* (Giarratana et al. 2017a); Mediterranean essential oils (*Origanum vulgare*, *Cuminum cyminum*, *Lavender stoechas*) (Pérez et al. 2017), *O. syriacum* (López et al. 2019), and *O. compactum* (López et al. 2018), – in saline solution, at different concentrations also exert in vitro activity against *Anisakis* larvae as well as the natural compound, allyl isothiocyanate (Giarratana et al. 2015). The monoterpenic derivatives obtained from different essential oils with larvicidal activity are *R* (+) limonene (Nalbone 2022) and α-pinene (Valero et al. 2015). The composition and activity of the most active plant extracts and essential oils were reviewed by Faria and Silva (2021). The potential application of these natural products against *Anisakis* larvae was demonstrated and for that reason recommended to industrial marinating seafood products processes. However, data on their toxicity and organoleptic properties is lacking (EFSA BIOHAZ Panel 2024b).

Photodynamic inactivation (PDI) can be an alternative treatment for *Anisakis* sp. control in fishery products. For photodynamic treatment to occur, three components are required: a photosensitizing molecule (PS) that acts as a photocatalyst, visible light and dioxygen (O_2_) (St. Denis et al. 2011). The PS is excited to a higher energy state by light, triggering a cascade of physical event (Youf et al. 2021). These events, in the presence of dioxygen in the ground state (^3^O_2_), lead to the production of various reactive oxygen species (ROS), such as radical species [e.g. superoxide anion radical (O₂⁻), hydroxyl radical (OH)] and singlet oxygen (^1^O_2_). The ROS, particularly ^1^O_2_, are primarily responsible for inducing cellular oxidative damage.

The role of porphyrins as PS in photodynamic treatment is well established, along with their photodynamic activity in the inactivation of several pathogens, including fungal pathogens (Dai et al. 2012), bacteria (St. Denis et al. 2011), viruses and parasites (Alouini et al. 2001). This includes tropical pathogens including Leishmania, *Trypanosoma brucei* (African trypanosomes) and *Plasmodium* spp. (Youf et al. 2021; Pinto et al. 2016). Others potential applications of porphyrins in PDI beyond the medical scope is agriculture plagues (Glueck et al. 2019; Hamminger et al. 2022), water disinfection (Almeida 2014, Bartolomeu 2024, Bonnet et al. 2006, Jemeli et al. 2002) including aquaculture waters (Alves et al. 2011, 2015; Arrojado et al. 2011; Bartolomeu et al. 2023; Fabris et al. 2012; Vieira et al. 2025). Over the past 15 years, significant research has been conducted to apply PDI across various fields. PS such as chlorophyllin were evaluated as a potential photodynamic substance in the presence of irradiation for ectoparasites in intensive aquaculture plants in fish parasites. Chlorophyllin showed efficacy of more than 50% in reducing the number of trophonts of the ciliated protozoan *Ichthyophthirius multifillis* (Richter et al. 2014). In other studies, the cationic 5,10,15,20-tetrakis(1-methylpyridinium-4-yl)porphyrin (TMPyP) exhibited biocidal activity against different pathogens but there is no evidence of the effect that this PS might have on parasite nematodes. Despite these advancements, no studies have yet explored the potential effect of PDI against foodborne parasites, such as L3 *Anisakis* larvae.

The objective of this study was: (1) to assess the efficacy of PDI on *Anisakis* larvae, using the cationic porphyrin, TMPyP as PS and (2) to understand how the selected PS inactivates *Anisakis* larvae.

## Materials and methods

### Photosensitizer and stock solution preparation

The cationic 5,10,15,20-tetrakis(1-methylpyridinium-4-yl)porphyrin tetraiodide (TMPyP) was obtained according to literature (Simões et al. 2016). Stock solutions of the TMPyP were prepared at 500 µM in dimethyl sulfoxide (DMSO) and kept in the dark. Immediately before each assay, the required TMPyP solutions were prepared in phosphate-buffered saline (PBS) at room temperature.

### Parasites collection

Live *Anisakis* larvae (type I, L3) were collected from fresh Atlantic horse mackerel, *Trachurus trachurus* purchased from a local market and provided from the Portuguese Coast. The larvae were obtained by dissecting specimens and visually inspecting the internal organs. The Atlantic horse mackerel was chosen because it is known to be frequently parasitized by nematodes of the genus *Anisakis* with a high prevalence and intensity of infection (Ramos 2020). Two batches of parasites were formed: (i) Batch A with larvae measuring ≈ 1.5 cm. A subsample was measured for this purpose, using a heat source to render the larvae unviable (*n* = 40; 2.12 ± 0.2 cm). The larvae were obtained from medium size fishes (35.5 ± 1.4 cm); (ii) Batch B with larvae measuring ≈ 2 cm. A subsample (*n* = 17) obtained from fish over 35 cm was measured for this purpose (1.5 ± 0.2 cm). Larvae (1.5 and 2.0 cm length) were placed in Petri dishes with NaCl 0.85% and visually assessed for viability, integrity and identified according to the macroscopic morphological features as L3 larvae of *Anisakis* type I. Larvae were handled with blunt-tipped needles to avoid damage and started moving with wriggling movements after being placed at room temperature. Only the L3 larvae actively moving and without any injuries were selected for treatment. In some assays, the collected larvae with ≈ 1.5 cm length were kept for 3 days in saline solution under refrigeration until the batch size for treatment was obtained. Larvae with ≈ 2 cm length were treated immediately after collected or maintained at 0.5 °C. Twenty larvae with vigorous movements were used for each treatment.

### *In vitro* treatments

Several experiments were conducted to determine the optimal PS combination of concentration and light irradiance under different microcosm conditions: (i) incubation of 15 min, 30 min, 1 h and 21 h; (ii) incubation temperatures of 0.5 °C (maintained in the fridge) and 22 °C (climate-controlled room); (iii) white light irradiation times of 2, 4, 6 and 8 h; (iv) light irradiance of 4, 14 and 67 mW/cm^2^ and TMPyP concentration of 50 µM prepared in phosphate-buffered saline (PBS). The selection of TMPyP concentration was based on earlier findings (Ramos et al. 2015, 2021, 2022b). All treatments were performed by immersing the larvae in plastic Petri dishes containing the TMPyP at the studied concentration. In each case, the experiments were conducted in triplicate using twenty larvae per sample. Light controls (larvae irradiated with white light in the absence of the TMPyP) and dark controls (larvae incubated with TMPyP at 50 µM without irradiation) were included in each assay. Larvae were exposed in PBS to 50 µM of TMPyP at different light irradiances (4, 14 and 67 mW/cm^2^) for 2, 4, 6 and 8 h. Prior light exposure, larvae were incubated in dark for 15 min, 30 min, 1–21 h at 0.5 or 22 °C in the presence of TMPyP. Larvae with an approximate length of 1.5 cm length (batch A) were used to optimise PDI parameters by testing multiple combinations of light irradiance and exposure time. Based on these results, larvae with an approximate length of 2.0 cm length (batch B) were exposed only to selected condition (TMPyP, 21 h of incubation, 67 mW/cm^2^ for 8 h PDI) to confirm the effects observed in batch A (Tables 1 and 2).

**Table 1:** Effect of different combinations of light irradiance, irradiation period and incubation time on gut destruction (%) and parasite viability in L3 *Anisakis* larvae from batch A in the presence of TMPyP at 50 µM after PDI

Light irradiance	Incubation time	Incubation temperature	Irradiation period	Parasite gut destruction (%)	Parasite viability score
4 mW/cm^2^	30’	22 °C	8 h	0 (*n* = 15)	3
14 mW/cm^2^	30’	22 °C	8 h	0 (*n* = 24)	3
	1 h		2 h	0 (*n* = 8)	3
			4 h	0 (*n* = 8)	3
			8 h	0 (*n* = 8)	3
67 mW/cm^2^	15’	22 °C	8 h	6.67 (*n* = 15)	3
	30’	22 °C	8 h	17.24 (*n* = 29)	3
	1 h	22 °C	8 h	19.60 (*n* = 51)	1
	21 h	22 °C	4 h	6.67 (*n* = 15)	3
		0.5 °C		6.67 (*n* = 15)	1
		22 °C	6 h	46.67 (*n* = 15)	0
		0.5 °C		66.67 (*n* = 12)	1 − 0
		22 °C	8 h	56.67 (*n* = 30)	0
		0.5 °C		50.00 (*n* = 16)	1 − 0

**Table 2:** Effect of incubation temperature on gut destruction (%) and parasite viability in L3 *Anisakis* larvae from batch B in the presence of TMPyP at 50 µM after PDI

Light irradiance	Incubation time	Incubation temperature	Irradiation period	Parasite gut destruction (%)	Parasite viability score
67 mW/cm^2^	21 h	22 °C	8 h	98.00 (*n* = 50)	0
		0.5 °C		95.23 (*n* = 20)	0

### Light source

The experiments were carried out using white light (400–700 nm) emitted by an El^®^MARK LED Panel, Type LED PANEL0303 (12 W, color temp: 4000–4500 K, China). To ensure accurate irradiance, a Coherent FieldMaxII-Top power meter, coupled with a Coherent PowerSens PS19Q energy sensor, was used to measure and adjust the light irradiance to 4, 14 and 67 mW/cm^2^.

### Larval viability assessment

When examining potential effects or responses of this nematode to PDI it was considered the motility/mobility of the larvae and morphological changes. Larval viability was assessed according to the modified score table of Hirasa and Takemasa (1998) by Guan et al. (2021). This table define the larval motility as “the in situ movement of different parts of the larvae body”. In turn, mobility was defined as “larval displacement inside the medium”. Accordingly, larvae with no motility at all were considered dead (score 0); larvae with motility only after stimulation by a needle were given a score 1; those with reduced motility were scored 2 and viable larvae were scored 3. During the experimental treatments at each fixed time interval, the larvae viability was checked at 2, 4, 6, and 8 h.

### Assessment of morphological changes

At the end of each assay, all the larvae were fixed in 10% buffered formalin and preserved in 70% ethanol for each treatment point, in dark conditions for further study. The histological study was done to assess larvae tissue morphological changes. Larval transversal sections were obtained (in dark conditions) and processed for histological examination. Sections with 3.0 μm thick were transversally cut through different levels. Haematoxylin-eosin (H&E) stained preparations were examined by microscope Olympus BX 51. Microtome cross-sections of non-treated and treated larvae were made to confirm changes caused by the treatments. The histological preparations were photographed using a Leitz Laborlux K light microscope (LM) connected to a Leica DFC 420 camera.

### Statistical analysis

Pearson correlation indexes were estimated to evaluate how the exogenous variable incubation time, irradiation time and temperature are related with morphological changes of the parasite. Additionally, a multiple linear regression model was adjusted using parasites (%) with morphological changes as the endogenous variable. All statistical analysis was performed using IBM SPSS Statistics v.23 (IBM Corp., Armonk, NY, USA) and R (R Core Team, 2021). Parasite intensity and abundance were estimated using Quantitative Parasitology (Reiczigel et al. 2019; on the web (version 1.0.15, 6 December 2020) by Jeno Reiczigel and Lajos Rozsa and web programming by Andras Reiczigel and Ibolya Fabian.

## Results

### Photodynamic inactivation assays in PBS solution

None of the L3 larvae from either batch or from the corresponding light and dark controls died during the incubation period, even at the longest incubation time (21 h) (data not shown) or in the PDI treatments performed with light irradiances of 4 or 14 mW/cm^2^. For both batches (A and B), *Anisakis* larvae mortality reached 100% (score 0) when, the larvae after 21 h of dark incubation at 22 °C, the larvae were exposed to white light for 6 h (only batch A) and 8 h (both batches) at an irradiance of 67 mW/cm^2^ (Tables 1 and 2).

In contrast, larvae that underwent the same white light exposure after 21 h of dark incubation at 0.5 °C exhibited a different response (Table 1). During the PDI treatment, some larvae died (score 0) or lost spontaneous motility (score 1) (Table 1). Although no mortality was observed following PDI treatment of 8 h of white light exposure at an irradiance of 67 mW/cm^2^ after dark incubation periods of 15 min, 30 min and 1 h at 22 °C, the histological sections of larvae from batch A revealed parasites with morphological changes in the intestinal wall (Table 1). For batch A, when the time of dark incubation, performed at both temperatures (22 °C or 0.5 °C) prolonged to 21 h followed by PDI treatment of 6 h and 8 h, the number of larvae with intestinal changes increased. In this batch A (Table 1), the percentage of larvae with gut changes depended on the irradiance conditions, but none or just small differences were observed between assays performed at the different incubation temperatures 22 °C or 0.5 °C. In batch B more than 95% of the larvae showed gut destruction (Table 2). In batch B, the percentage of larvae with gut changes was similar at 22 °C and 0.5 °C with a higher mortality (score 0) and a more exuberant cytotoxic effect, resulting on severe destruction of the intestinal epithelium than the larvae in batch A at the same conditions (Table 1). A multiple linear regression model was fitted using the number of parasites with gut destruction as the dependent variable, and the incubation time, irradiation period and incubation temperature as independent variables. ​In the initial model, incubation time and irradiation period showed statistically significant associations with the percentage of parasites with gut destruction, whereas incubation temperature did not reach statistical significance and was therefore excluded from the final model. The final adjusted model (Model statistics: $\:{R}^{2}=0.69$, adjusted $\:{R}^{2}=0.64$, F(2,12) ≈ 13.5, p-value ≈ 0.0009) indicates that incubation time and irradiation period together explain about 69% of the variance in the number of parasites with gut destruction.​ Although the sample size is small, the estimated regression coefficients suggest that, on a per-hour basis, irradiation period contributes several times more to the number of parasites with gut destruction than incubation time (approximately 6–7-fold difference in effect size). Additional experiments with a larger number of larvae and a wider range of treatment conditions will be required to confirm these preliminary findings and to clarify the remaining 31% of variability in the number of parasites with gut destruction. The direct comparison between larvae measuring 1.5 cm and 2.0 cm is only possible under identical experimental conditions, specifically the same incubation time (21 h) and white light irradiation period (8 h). Therefore, the analysis between lengths was limited to the last treatment combination of batch A (1.5 cm) (Table 1) and batch B (Table 2), which share exactly these experimental parameters. Outside of this common combination, any differences observed could be due to both the larval length and differences in incubation time or white light irradiation period; thus, the effect cannot be solely attributed to length. If, under these specific conditions (21 h incubation with TMPyP, 8 h of white light irradiation), a difference in destruction percentage is observed between 1.5 cm and 2.0 cm larvae, it suggests that length may influence susceptibility to treatment; however, further tests are needed to confirm this effect. With very few observations (likely 2 to 4 per group/combination based on the total of 15 assays from the previous analysis), formal tests such as t-test, ANOVA or regression with interaction (length × irradiation) have very low statistical power and a high risk of false negatives. With such limited replicates, significance tests are not very informative.

### Microscopic observation of the larvae

#### Control larvae

**Fig. 1:**
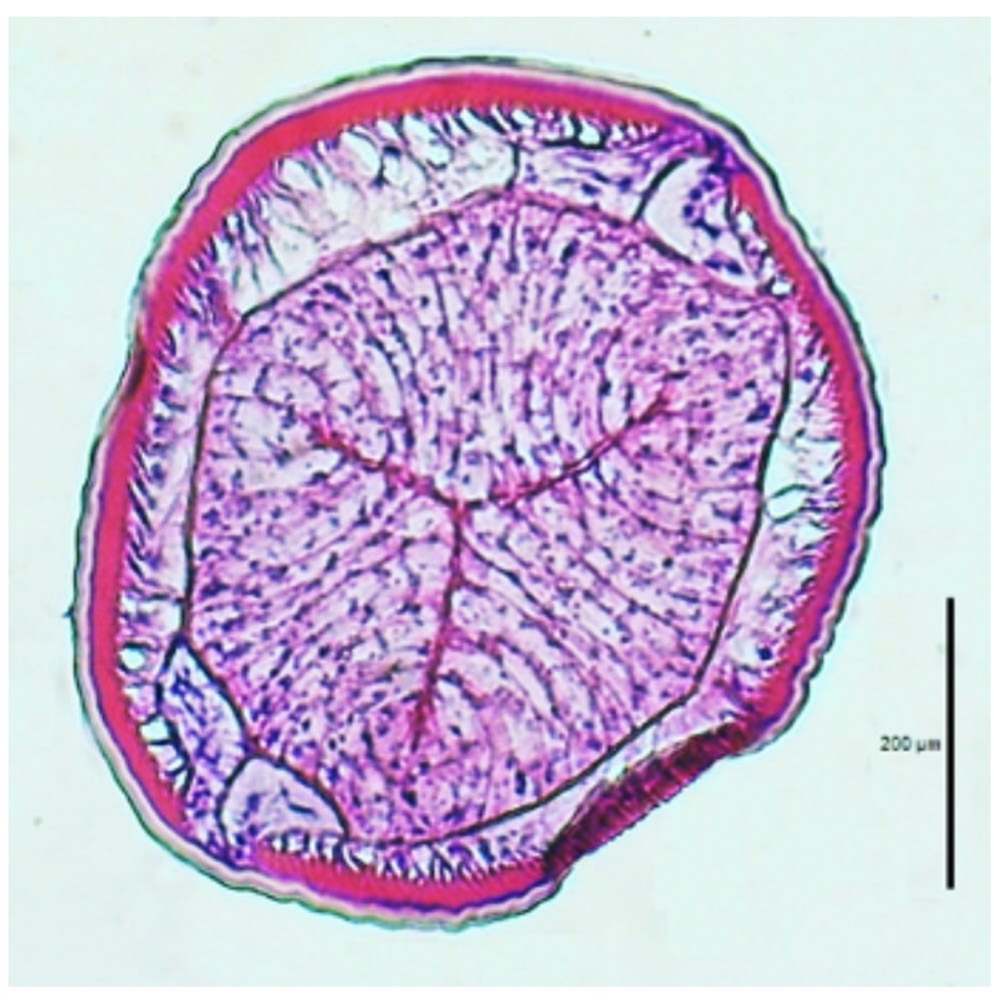
Light micrograph of a histological cross section through the intestinal region of the non-treated L3 *Anisakis* larvae

All examined larvae belonged to the *Anisakis* genus. Under light microscopy, the alimentary tract of L3 larvae displayed the general structure of nematode gut, as described by Grabda (1976) (Fig. 1).

Sections of the anterior part of the larvae showed the morphological structures of the third-stage larvae of *Anisakis*, the ventriculus (glandular oesophagus) at the esophago-intestinal junction. Cross sections through intestinal region of the L3 *Anisakis* larvae reveal the external cuticle overlying the celomyarian and polymyarian muscle layer with the dorsal and ventral epidermal cords and the Y-shaped lateral epidermal cords. The intestinal epithelium consists of a monolayer of tall columnar epithelial cells, which determine a central tripartite intestinal lumen. These cells have a granulated cytoplasm and the nuclei is located basally near the intestine basal membrane; the apical membrane is covered by a thin microvillus layer. Along the anterior part of the intestine, the unpaired excretory gland (renette gland) was observed. No reproductive system was present. The posterior end of the gut terminates with a mucron.

### PDI treated larvae

**Fig. 2:**
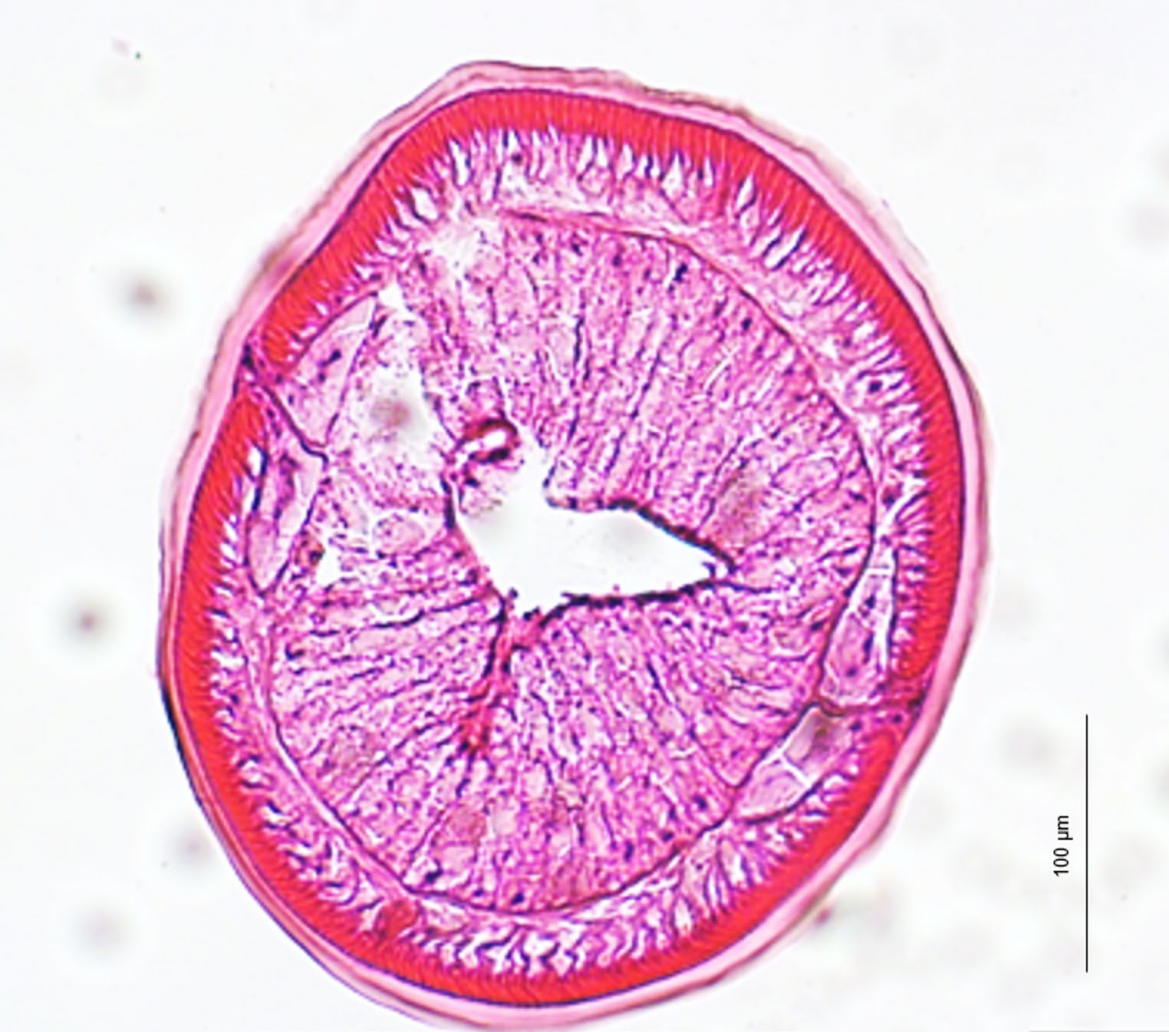
Representative light micrograph of a histological cross-section of the intestinal region of L3 *Anisakis* larvae of batch A after PDI treatment. Larvae were incubated with TMPyP for 15 min, 30 min, 1h or 21 h at 22 °C and irradiated with white light at 67 mW/cm^2^

The histopathological study revealed larvae without changes in the intestinal wall (at a light irradiance of 4 mW/cm^2^ and 14 mW/cm^2^) and evolutionary degrees of morphological changes of the intestinal wall (at an irradiance of 67 mW/cm^2^) (data not shown). The histological sections of larvae from batch A following PDI treatment with 8 h of white light exposure at an irradiance of 67 mW/cm^2^ after dark incubation periods of 15 min, 30 min and 1 h at 22 °C, revealed compression of the epithelial intestinal cells with loss of their cylindrical shape (Fig. 2). The intestinal lumen was enlarged and no longer exhibit its triradiate appearance. Usually, one of the rays was distinctly distended (Fig. 2).

**Fig. 3:**
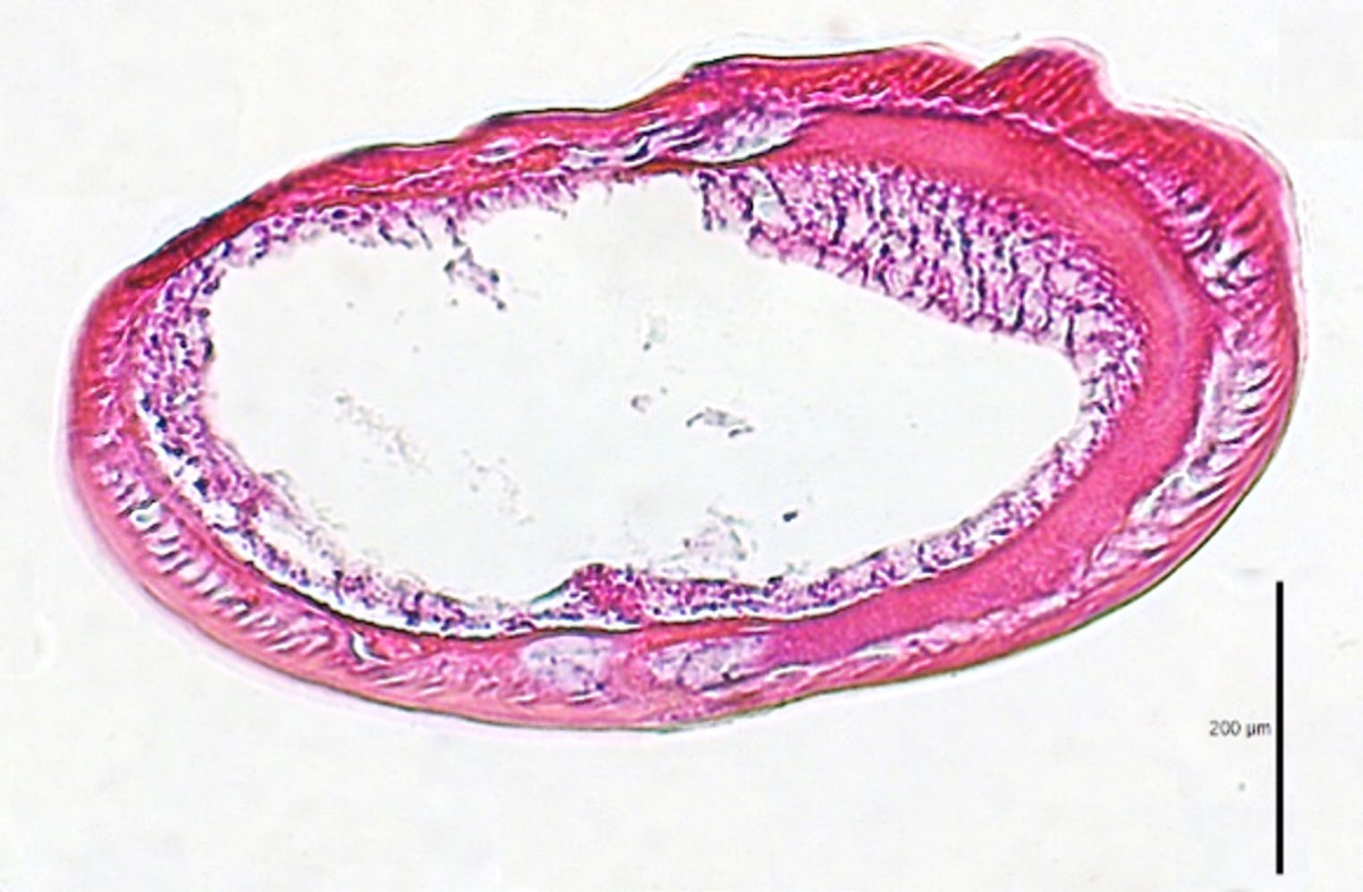
Representative light micrograph of gut destruction observed in L3 *Anisakis* larvae of batch B after PDI treatment. Larvae were incubated with TMPyP at 0.5 or 22 °C and irradiated 8 h with white light at 67 mW/cm^2^

The more exuberant gut destruction was observed in batch B (larvae with 2.0 cm) (Fig. 3). Cross sections through the intestinal region of the treated L3 *Anisakis* larvae revealed destruction of the intestinal wall towards the outside (cuticle), necrosis of the epithelial cells of the intestine, broadening of the intestinal lumen with the presence of cellular material (Fig. 3).

## Discussion

The effectiveness of PDI in *Anisakis* L3 larvae inactivation and its mechanism of action, were provided by the results obtained from observing the larval behavior under various treatment conditions, as well as the corresponding morphological changes.

Data show that under specific treatment conditions, the small body section and transparency of L3 *Anisakis* larvae allowed the successful use of PDI approach, resulting in larvae death. Previous assays (Ramos et al. 2015, 2021) confirmed the larvicidal effects of TMPyP toward L3 *Anisakis.* The higher mortality was observed in larvae with 2.0 cm in length, which was associated with intestinal damage observed after PDI treatment. The L3 larvae was a non-feeding stage (Grabda 1976) and the intestinal damages observed in the histological study supports the possibility of the TMPyP present in the aqueous medium entering *per os* without damaging the cuticle. To improve passive exposure of the larvae to the TMPyP solution within the digestive tract, the effect of the “incubation temperature” as a physical stimulus on L3 larvae motility was assessed. Two incubation temperatures were selected (a) 22 °C, which promotes high mobility and motility of *Anisakis* larvae compared with low temperatures (Guan et al. 2021) and (b) 0.5 °C, as refrigeration below 2 °C inhibits *post-mortem* migration (Cipriani et al. 2016).

Increased larvae movement at 22 °C did not necessarily result in greater exposure to the TMPyP solution. According to Guan et al. (2021), although the larvae movement increases at 22 °C, the duration and intensity of these movements vary among individuals, which may influence the PS intake *per os* by passive transport. In the present study, increasing the dark incubation period to 21 h was associated with enhanced photodynamic effects and subsequent white-light irradiation for 6 and 8 h resulted in 100% mortality in both batch A and batch B. The irradiation period parameter contributes approximately 6–7-fold more to the observed intestinal changes than the incubation time. During the irradiation period, larvae remained immersed in the TMPyP solution, allowing exposure throughout light treatment. The increased effect observed after longer incubation and irradiation periods may therefore reflect prolonged exposure to the PS.

All larvae analyzed were at the L3 stage, however, some variability in larvae size was observed. Data show greater resistance of small *Anisakis* L3 (batch A with 1.5 cm) than the longer ones (batch B with 2.0 cm) to the PDI treatments. The results showed a high variability in parasite responses (parasite percentage with gut destruction and parasite viability score at different irradiance) in the same treatment (batch A with 1.5 cm) and between treatments (batches A and B), which could be due to the degree of morphological changes of larvae. On the other hand, each trial exhibited variable intestine wall changes, more observed in batches with 1.5 cm larvae length, which means the amount of PS in the intestine might be different. This variability may reflect differences in individual behaviors in the resistance of L3 *Anisakis* to oxidative stress induced by PDI treatment, and in the encysted state from which larvae were removed prior to treatment. The photoinduced effects was associated with incubation time, irradiation duration and PS distribution accounting for intestinal damage observed in approximately 69% of the larvae. The remaining larvae with intestinal changes (approximately 31%) may be related to inter-individual differences, including larvae size or pre-treatment encysted state, however, no clear relationship between larvae length and response to PDI treatment was established. Notably, different responses were observed among individuals within the same batch, reinforcing the presence of intrinsic inter-individual variability. Data show the efficacy of the PDI treatment was related to the larva size, since it was registered 100% mortality in batch B with larvae with 2.0 cm length. Several studies demonstrated that the wide variability of anisakids larvae responses to marinating and thermal treatments could be related also to the larval size (Giarratana et al. 2012). The nematocidal activity of several essential oils and their components were tested with selected L3 larvae over 2.0 cm in length (Giarratana et al. 2012; Gómez-Rincón et al. 2014; Lin R-J et al. 2010; López et al. 2015, 2018; Navarro et al. 2008; Romero et al. 2012, 2014). In this batch B, with longer larvae, the cytotoxic effect resulted in the complete destruction of enterocytes, meaning that the LD50 could be obtained with shorter incubation time and irradiation period and lower PS concentration, which requires further experimental trials.

The *Anisakis* L3 represent a non-feeding stage and for this reason, the collapsed intestine present in cross-sections, a triradiate lumen lined with a monolayer of cellular epithelium. During the treatments, it was observed the occurrence of morphological changes in the enterocytes, which lose their cylindrical appearance, in different evolutionary stages of destruction of the intestinal wall. The larvae from batch A, subjected to PDI treatments of 8 h of white light exposure at an irradiance of 67 mW/cm^2^, after dark incubation periods of 15 min, 30 min and 1 h at 22 °C, showed distension of one of the intestinal lumen rays, without, however, losing its triradial aspect. When batches A and B were irradiated with 67 mW/cm², after 21 h of incubation and 8 h of exposure to white light, destruction of the cell wall was observed, with necrosis of the enterocytes, resulting in the loss of the triradial appearance of the intestine. The TMPYP intake leads to the photodynamic inactivation of the L3 *Anisakis* due to the direct action of the ROS in the target intestinal cells, which is strengthened by the morphological intestinal changes observed. Thus, the photodynamic action of TMPyP is responsible for enterocytes destruction and the killing of L3 *Anisakis* larvae. The histopathological study showed damages of digestive tract but there was no evidence of cuticle damages. However, cuticle and digestive tract damages were observed in parasites treated with natural compounds (Giarratana et al. 2014, 2015, 2017; Navarro et al. 2008; Romero et al. 2012; Trabelsi et al. 2019; Valero et al. 2015) and affected chords of larvae were observed too (Navarro et al. 2008). Authors emphasized the role of lipophilia in the biocidal action exerted by terpenic components of essential oils, which modifies the permeability and integrity of membrane channels, resulting in cellular damage (Giarratana et al. 2014; Navarro et al. 2008). However, in this study, the undamaged cuticle forms an impermeable barrier between the larval body and the PS medium preventing its diffusion across larvae body. Bypassing the *Anisaki*s cuticle presents a challenge due to the multi-layered constituents of larval cuticle, which are composed of insoluble proteins (cuticlins), glycoproteins, collagen and lipids (Page and Johnstone 2007). The TMPyP action in the intestinal cells, without cuticle damage, allows the retention of PS after photodynamic action in larvae. This feature is important because it prevents the possible diffusion of products resulting from PDI, and ensures that the parasite’s allergenic proteins do not release.

Different factors as the origin, age and physical conditions of larvae (batch effect) may influence larval behavior and their movements (Guan et al. 2021). In these treatments the potential batch effect was accounted only for the longer larvae (batch B), which were obtained from two Atlantic horse mackerel with a high *Anisakis* infection. The great variability observed in the treated batch A may be due to size variability and physical conditions of the larvae which were obtained from different hosts purchased in different days, with some of them being encysted. In the present study, incubation temperature of 0.5 °C and 22 °C under dark conditions seems to have no relevant influence on the intake of TMPyP into the larvae. The passive transport of the PS from the medium into the larvae body is strongly enhanced with the increase of incubation time. On the other hand, by increasing the incubation time with prolonged exposure, the irradiation period contributed several times more to the number of parasites with intestinal destruction than the incubation time (a difference of approximately 6 to 7 times in the effect size), since we consider that prolonged exposure promotes greater absorption of PS by passive transport.

## Conclusions

This work is the first study concerning in vitro effect of PDI against the fish parasite *Anisakis* L3 larvae and enhanced our knowledge of the photobiological effect of PDI on metazoan, specifically the nematode.

Data suggest PDI is able to devitalize L3 *Anisakis* larvae after exposure to the TMPyP solution and subsequent irradiation under different conditions, but their behaviour is influenced by larvae size –physical condition and encysted state of larvae. The larvicidal activity is probably related to the damage found in the parasite digestive tract. According to data, PDI can be considered as an alternative option to freezing to inactivate L3 *Anisakis* larvae. Since some porphyrin derivatives have been authorized by the EC Regulation in the food industry as additives, such as chlorophyllin (E170) (Richter et al. 2014) it allows exploring their use in the prevention of anisakiasis. Further studies are required to investigate whether PDI of seafood products with these type of PS could have a prophylactic effect, reducing the pathogenicity of L3 *Anisakis* type I in humans. Overall, the results should be regarded as a proof of concept, indicating the potential of photodynamic inactivation as a non-destructive approach; however, the method remains exploratory, and substantial further research is required before any practical application can be considered.

## Data Availability

Data are available upon request from reviewers. After manuscript acceptance, they will be made available in a repository.
